# Screening for maternal cytomegalovirus infection during pregnancy and pregnancy outcome in patients with liver disease: an observational study

**DOI:** 10.1186/s12879-023-08144-9

**Published:** 2023-04-06

**Authors:** Rasha Eletreby, Rasha Abdelaziz, Hend Ibrahim Shousha, Zeinab Hammam, Ayman Hany, Dina Sabry, Basma Elawady, Naglaa Zayed, Ayman Yosry, Shereen Abdel Alem

**Affiliations:** 1 Endemic Medicine and Hepatology Department, Faculty of Medicine, Cairo University, Cairo, Egypt; 2 Gynecology and Obstetrics Department, Faculty of Medicine, Cairo University, Cairo, Egypt; 3 Medical Biochemistry and Molecular Biology Department, Faculty of Medicine, Cairo University, Cairo, Egypt; 4 Medical Microbiology and Immunology Department, Faculty of Medicine, Cairo University, Cairo, Egypt

**Keywords:** Cytomegalovirus, Liver disease, Screening, Pregnancy, Outcome

## Abstract

**Background:**

Cytomegalovirus (CMV) infection among pregnant females could induce CMV hepatitis with possible changes in liver stiffness measurement (LSM) which could be reversibly increased during normal pregnancies, particularly in the third trimester. This study aimed to detect the prevalence of CMV infection among pregnant females with and without chronic liver disease and to evaluate the effects of CMV infection on LSM and pregnancy outcomes in comparison to non-CMV-infected pregnant females.

**Methods:**

This is an observational prospective study that included 201 pregnant ladies presented to the liver disease with pregnancy clinic, Cairo University from March 2018 to April 2019. We assessed the laboratory results, abdominal ultrasonography, LSM using ARFI elastography, and pregnancy outcomes.

**Results:**

Two hundred and one pregnant ladies were divided into ; group 1: pregnant ladies with normal pregnancy (n = 128), group 2: pregnant ladies with chronic liver diseases not related to pregnancy (n = 35), and group 3: pregnant ladies with pregnancy-related liver diseases (n = 38). Positive CMV serology (either/or, +ve CMV-IgM, IgG) was detected in 106/201 patients (52.74%), and fifteen of them had an active infection (IgG +, IgM+, PCR+). Pregnant females with chronic liver diseases not related to pregnancy had significantly higher serum levels of CMV IgM, IgG, and PCR. Moreover, LSM had a significant correlation with CMV IgG and CM_PCR in normal pregnant ladies. Maternal mortality occurred only in pregnant females with chronic liver diseases in 5.7% (2/35).

**Conclusion:**

Maternal CMV infection carries a significant risk to pregnant females with chronic liver disease. Routine CMV screening for women planning to be pregnant, especially those with chronic liver disease could help to avoid bad maternal and fetal outcomes.

## Background

Cytomegalovirus (CMV) is a DNA herpes virus characterized by latent infection. Although CMV infection is generally asymptomatic, it can cause severe disease, especially in immune-compromised patients, transplant recipients, and newborn neonates [1]. Maternal CMV infection in the first trimester of pregnancy is a public health concern and the most common congenital viral infection. Congenital infection may be asymptomatic or symptomatic; symptomatic disease occurs in about 10% of neonates at birth and can be severe and life-threatening [2]. Congenital cytomegalovirus infection (c CMV) is the main cause of hearing loss and mental retardation in infants without genetic disorders [3].

Seronegative females for CMV antibodies among middle and higher socioeconomic status could easily acquire primary CMV during a child-bearing period and transmit the virus to their babies as they are less likely to produce neutralizing antibodies [4] Moreover, pregnant women with their immune downregulation are particularly at risk of CMV reactivation [5]. Pregnancy did not appear to affect the clinical severity of CMV in different studies [6]. CMV hepatitis in immune-competent adults is usually asymptomatic with mild transaminitis. Bilirubin can be completely normal or only mild-to-moderately elevated [7], However, CMV infection is associated with poor outcomes in patients with chronic liver disease (CLD) especially liver cirrhosis. In a study done by Faivre et al., in 2019, on 1178 cirrhotic patients, CMV-seropositive cirrhotic patients were at higher risk of liver-related death caused by severe cirrhosis complications or more aggressive HCCs [8]. Elastography values may be falsely high when blood flow to the liver is increased, as during the late stages of pregnancy [9]. Ribeiro and colleagues found that liver stiffness (LS) and controlled attenuation parameters (CAP) increase reversibly during normal pregnancies and that slightly elevated levels in the third trimester can be considered a normal finding [10].

The current study aims to evaluate the prevalence rate of CMV infection among a cohort of Egyptian pregnant females presented to a tertiary hospital and to evaluate the subsequent effects of CMV infection on liver stiffness measurement and pregnancy outcomes in comparison to non-CMV infected healthy pregnant females and whether the presence of underlying liver disease (either pregnancy related or not) may alter CMV effects on liver stiffness measurement (LSM) and/or pregnancy outcomes.

## Patients and methods

### Study population

This is an observational prospective study that included 201 pregnant ladies presented to the liver disease with pregnancy clinic, Endemic Medicine Department and Obstetric outpatients’ clinic, Faculty of Medicine, Cairo University in the period from March 2018 to April 2019. Pregnant females were assigned into three groups; group 1 including pregnant women with normal pregnancy (n = 128), group 2: pregnant women with CLD not related to pregnancy such as chronic viral hepatitis, autoimmune liver disease,….etc (n = 35), and group 3: pregnant women with liver diseases associated with pregnancy such as intrahepatic cholestasis of pregnancy and hemolysis, elevated liver enzymes and low platelets count (HELLP) syndrome (n = 38).

All patients gave informed consent including the study procedures and approved the usage of blood sampling and possible data application in future research. The study protocol was approved by the ethics committee of the Faculty of Medicine, Cairo University number: I-251,017. The study was also as part of the project entitled “non-invasive diagnosis and prediction of progression of hepatic fibrosis and related complications using biochemical, molecular, gentic and imaging methods in chronic Egyptian patients; project ID 5274; approval ethical code N-53-2014” that funded by STDF.

Inclusion criteria included pregnant women ≥ 18 years with or without liver diseases, while participants with missed abortions (negative cardiac pulsation) or those who refused to participate in the study were excluded.

According to CMV infection status, pregnant women were further classified into:

1. Seronegative for CMV infection (IgG -, IgM-, PCR-) (n = 95).
2. Seropositive for CMV infection (n = 106). Those also were sub-classified into:
  Previous or past CMV infection (IgG+, IgM-, PCR-).
  Chronic CMV infection (IgG+, IgM-, PCR+).
  Recent CMV infection (IgG+, IgM+, PCR-).
  Current or active CMV infection (IgG +, IgM+, PCR+) [11].

### Clinical assessment

#### a- History taking:

demographic features, presence of co-morbidities, and a history of past maternal and fetal complications (medical complications during a previous pregnancy, abortion, preterm labor, stillbirth, newborn with congenital anomalies). Possible risk factors for exposure to CMV infection e.g. risk of parenteral exposure, previous history of organ transplantation, or other children in the family with Daycare centers. Manifestations suggesting CMV infection e.g. intermittent fever, chills, sore throat, loss of appetite, and/or jaundice. and manifestations of hepatic decompensation were also reported.

#### b- Routine antenatal care involving:

**1st US fetal scan** at 1st visit at antenatal care obstetric clinic for detection of number and site of the gestational sac.

**1st -trimester anomaly US scan** between 11 and 13 weeks of gestation.

**22nd weeks fetal US scan** (2nd trimester scan) to detect any congenital anomalies seen in the fetus.

#### c- Outcomes:

Maternal and fetal outcomes in the form of morbidity, mortality, and adverse pregnancy outcomes such as preterm labor and abortion were recorded.

### Laboratory investigation

Complete blood count, liver biochemical profile: Alanine transaminase (ALT), Aspartate transaminase (AST), Alkaline phosphatase (ALP), serum albumin, total bilirubin, INR; viral hepatitis markers (HBsAg, HCV Ab) by ELISA technique.

### CMV testing

Cytomegalovirus IgG and IgM were determined by ELISA kits. Cut-off values of 1.2 IU/mL and 1.32 IU/mL were considered reactive for CMV IgG and CMV IgM, respectively. Samples with positive CMV serology were further checked by quantitative real-time polymerase chain reaction (RT-PCR) for quantitation of CMV-DNA [12–14].

### Acoustic radiation force impulse imaging elastography (ARFI)

ARFI was done for 130 pregnant women to assess liver fibrosis using a Siemens ACUSON S3000 Ultrasound System (Siemens AG, Erlangen, Germany) with a 6C1 HD transducer, by using Virtual Touch Tissue Quantification (VTTQ) application according to the manufacturer’s instructions [15]. Out of 130 pregnant women, 80 of them were normal pregnant women, 27 of them had liver disease unrelated to pregnancy, and 23 with pregnancy-related liver disease. Follow-up by ARFI was done for 114 women after delivery.

## Statistical methods

The patient’s quantitative data were expressed by the mean and standard deviation (SD). They were compared by paired and unpaired t-student tests. A nonparametric correlation was done to correlate nonparametric quantitative data. Qualitative data were expressed by number and percentage. They were compared by the Chi-square test and Wilcoxon signed-rank test. In all tests, the p-value was considered significant if less than 0.05.

## Results

### Study cohort

This study included two hundred one pregnant ladies with healthy liver and those with liver disease either related or non-related to pregnancy attending routine antenatal care. Pregnant women were further classified according to CMV infection status into those who were seronegative for CMV infection (n = 95), and those who had either past CMV infection (n = 73), chronic CMV infection (n = 9), recent CMV infection (n = 9) and current or active CMV infection (n = 15) as shown in (Table 1).

**Table 1:** Patients’ grouping and stratification according to liver disease status and CMV infection status

			Count (%)
**Group**	**1**	Normal pregnant	128 (63.7%)
	**2**	Pregnant with liver diseases not related to pregnancy	35 (17.4%)
	**3**	Pregnant with pregnancy associated liver disease	38 (18.9%)
**Group 2 subgroups**		Chronic hepatitis C	19 (54.3%)
		Chronic hepatitis B	8 (20.0%)
		Autoimmune hepatitis	3 (8.6%)
		Other diseases as Budd-Chiari syndrome, Wilson disease, Cigler-Najjar syndrome Gaucher disease, and acute hepatitis E.	5 (17.1%)
**Group 3 subgroups**		Preeclampsia	24 (63.2%)
		Intrahepatic cholestasis of pregnancy	6 (15.8%)
		Hyperemesis gravidarum	8 (21.1%)
**Stratification according to presence or absence of CMV infection**		No CMV infection	95 (47.26%)
		Pregnant with CMV infection	106 (52.74%)
**Type of CMV infection (n = 106)**		Previous or past CMV infection	73 (68.87%)
		Chronic CMV infection	9 (8.49%)
		Recent CMV infection	9 (8.49%)
		Current or active CMV infection	15 (14.15%)

### CMV infection among the studied patients

Serological evidence of CMV infection among healthy pregnant females and those with liver disease is shown in (Table 2). The mean serum levels of CMV IgM, IgG, and DNA by quantitative PCR were significantly higher among the group of pregnant females with chronic liver diseases not related to pregnancy.

**Table 2:** Frequency of positive serological tests for CMV infection and CMV PCR among all study participants (n = 201)

			Normal pregnant women (n = 128)	Pregnant women with liver diseases not related to pregnancy (n = 35)	Pregnant women with pregnancy associated liver disease (n = 38)	P-value
Serological tests for CMV	CMV IgG	Count (%)	53^a^ (41.4%)	27^b^ (77.1%)	24^b^ (63.2%)	**< 0.001**
		Titers (IU/mL) (mean ± SD)	0.95 ± 0.61^a^	1.44 ± 0.68^b^	1.37 ± 0.98^b^	**< 0.001**
	CMV IgM	Count (%)	8^a^ (6.3%)	11^b^ (31.4%)	6^ab^ (15.8%)	**< 0.001**
		Titers (IU/mL) (mean ± SD)	0.55 ± 0.38^a^	0.90 ± 0.56^b^	0.644 ± 0.45^a^	**0.002**
PCR assay for CMV	CMV DNA- based PCR	Count (%)	4^a^ (7.5%)	15^b^ (55.6%)	5^a^ (20.8%)	**< 0.001**
		Titers (IU/mL) (mean ± SD)	126.40 ± 723.63^a^	58262.48 ± 182255.34^b^	136.00 ± 288.23^a^	**< 0.001**

Data are shown in n (%) and mean (SD)

Statistically significant values are in bold

History of previous abortion, preterm labor, DM, and being multigravida were the significant factors for having higher CMV IgG titers in ladies with normal pregnancy, however, none of these baseline factors were significantly associated with positive CMV PCR values (i.e. active CMV infection). On the other hand, in the pregnancy-associated liver disease group; patients with hypertension or a history of previous abortion had higher CMV IgG titers. Among pregnant ladies with chronic liver diseases not related to pregnancy; CMV IgM titers were higher in those with a history of previous abortion. In either group of liver disease (groups 2 and 3), none of the baseline factors was significantly related to having active CMV infection apart from being in the second trimester for group 3 (Table 3).

**Table 3:** Relation between serological tests of CMV infection and baseline demographic features among all study participants (n = 201)

					CMV IgG (IU/mL)	CMV IgM (IU/mL)	CMV PCR (IU/mL)
**Group 1** **(Normal pregnancy; n = 128**)	Primigravida				0.47 ± 0.19	0.42 ± 0.34	BDL
	Multigravida				1.02 ± 0.62	0.57 ± 0.38	126.40 ± 723.63
	*p-*value				***0.002***	0.09	……
	Previous abortion			Yes	1.37 ± 0.56	0.50 ± 0.30	128.53 ± 796.89
				no	0.62 ± 0.42	0.60 ± 0.43	117.20 ± 248.64
	*p-*value				***< 0.001***	0.36	0.101
	Preterm labor			Yes	1.48 ± 0.48	0.49 ± 0.23	BDL
				no	0.91 ± 0.61	0.56 ± 0.39	148.87 ± 784.49
	*p-*value				***0.007***	0.98	0.71
	Diabetes mellitus		Yes		1.46 ± 0.19	0.71 ± 0.33	746.29 ± 1974.49
			no		0.92 ± 0.62	0.54 ± 0.38	32.07 ± 128.09
	*p* value				***0.028***	0.109	0.729
**Group 2** **(Pregnant with chronic liver diseases unrelated to pregnancy; n = 35)**	Previous abortion		Yes		1.40 ± 0.39	0.51 ± 0.24	BDL
			no		1.46 ± 0.77	1.05 ± 0.58	87393.72 ± 219332.71
	*p* -value				1.000	***0.013***	***< 0.001***
**Group 3** **(Pregnant with pregnancy-associated liver disease ;n = 38)**	Trimester		First		1.19 ± 0.56	0.56 ± 0.44	BDL
			Second		1.52 ± 1.37	0.74 ± 0.54	296.73 ± 372.32
			Third		1.31 ± 0.59	0.58 ± 0.37	BDL
	*p-*value				0.854	0.67	0.03
	Previous abortions	Yes			1.91 ± 1.11	0.73 ± 0.44	98.39 ± 290.38
		no			0.89 ± 0.49	0.57 ± 0.46	248.83 ± 274.05
	*p-*value				***< 0.001***	0.15	0.25
	Hypertension	Yes			1.70 ± 1.24	0.58 ± 0.38	53.21 ± 199.11
		no			1.08 ± 0.54	0.70 ± 0.51	251.90 ± 359.81
	*p-*value				***0.04***	0.52	0.21

Data are shown in mean+/- SD

BDL: below detection limit

Statistically significant values are in bold

### ARFI elastography

**Fig. 1:**
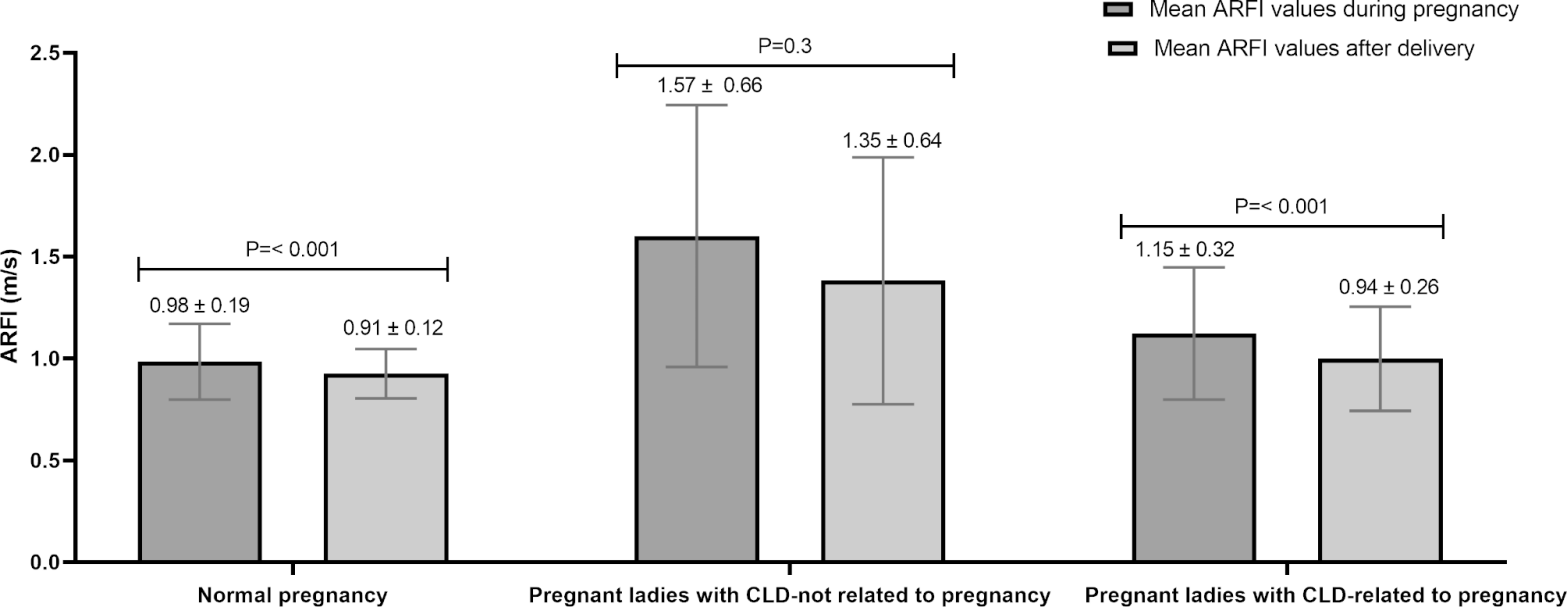
Mean LSM values by ARFI elastography in the studied groups

ARFI was successfully done in 130 study participants during pregnancy and 114 after delivery to assess liver stiffness measurement. Baseline and post-delivery mean ARFI results were higher among pregnant ladies with chronic liver diseases not related to pregnancy and did not show significant changes after delivery (1.57 vs. 1.35 m/s, p = 0.3). On the other side, mean ARFI results of ladies with normal pregnancy and ladies with liver diseases related to pregnancy significantly decreased after delivery (0.98 vs. 0.91 m/s, p = < 0.001; 1.15 vs. 0.94 m/s, p = < 0.001) (Fig. 1).

**Fig. 2:**
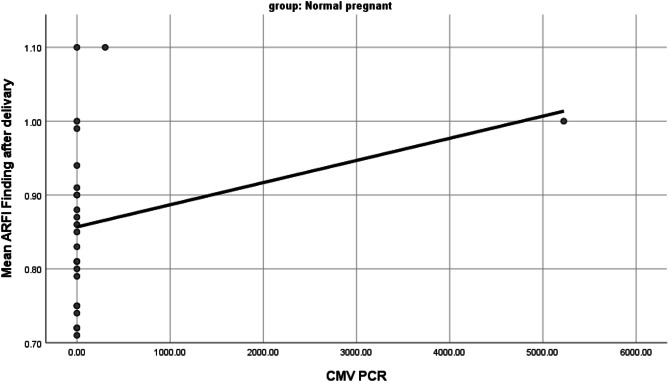
Correlation between ARFI values and CMV serology and PCR levels

We further studied the correlation between ARFI results and CMV serology and PCR levels of the studied patients. The only significant correlation was within ladies with normal pregnancy where there was a negative significant correlation between ARFI results and CMV IgG (r= -0.4, p = 0.002) and a positive significant correlation between ARFI results and CMV PCR(r = 0.43, p = 0.03) reflecting that liver stiffness by ARFI increases with the increase in CMV viral load as measured by PCR (Fig. 2).

### Pregnancy outcomes

Pregnancy outcome (both maternal and fetal) according to CMV infection and liver disease status is shown in Table 4. Four pregnant females with CMV infection had preterm labor, stillbirth occurred in 3 cases, fetal congenital heart disease in one case, and maternal mortality in two cases. Maternal mortality occurred only in pregnant females with chronic liver diseases not related to pregnancy; severe vascular decompensation in HBV-related liver cirrhosis and HEV-related fulminant liver failure were the causes of maternal mortality.

**Table 4:** Pregnancy outcomes according to CMV infection and liver disease status

		CMV infection status		Liver disease status		
		Pregnant with no CMV infection (n = 95)	Pregnant with CMV infection (n = 106)	Normal pregnant (n = 128)	Pregnant with liver diseases not related to pregnancy (n = 35)	Pregnant with pregnancy-associated liver disease (n = 38)
**Pregnancy outcome, n (%)**	**Normal**	86 (90.53%)	86 (81.13%)	120^a^ (93.8%)	26^b^ (74.3%)	27^b^ (71.1%)
	**Abnormal**	9 (9.47%)	20 (18.87%)	8^a^ (6.3%)	9^b^ (25.7%)	11^b^ (28.9%)
**P-value**		**< 0.001**		**< 0.001**		
**Pregnancy outcome details**, **n(%)**	**Normal**	86 (90.53%)	86 (81.13%)	120^a^ (93.8%)	26^b^ (74.3%)	27^c^ (71.1%)
	**Abortion**	0^a^ (0.0%)	1 (0.94%)	0^a^ (0.0%)	1^b^ (2.9%)	0^c^ (0.0%)
	**Preterm labor**	4 (4.21%)	11 (10.38%)	3^a^ (2.3%)	2^b^ (5.7%)	10^c^ (26.3%)
	**Still-birth**	3 (3.16%)	3 (2.83%)	3^a^ (2.3%)	1^b^ (2.9%)	1^c^ (2.6%)
	**Congenital anomalies**	1 (1.05%)	4 (3.77%)	2^a^ (1.6%)	3^b^ (8.6%)	0^c^ (0.0%)
	**Maternal mortality**	1 (1.05%)	1 (0.94%)	0^a^ (0.0%)	2^b^ (5.7%)	0^c^ (0.0%)
**p-value**	**0.001**			**0.001**		

Data are shown in n (%)

Statistically significant values are in bold

## Discussion

Pregnancy has been described as an immunological condition that presents multiple challenges in the diagnosis, prevention, and management of infectious diseases. Maternal CMV is usually asymptomatic and is the most common cause of congenital infection. However, the presence of chronic liver disease in pregnant females may expose them to a higher risk of liver-related death in CMV-seropositive cirrhotic patients [8]. Routine screening for CMV infection among pregnant ladies is not recommended. Thus, the evaluation of maternal CMV seroprevalence and the possible effects on maternal hepatic abnormalities and fetal outcomes is mandatory.

In the current study, 106 (52.7%), 24 (11.9%), and 15 (7.46%) patients had positive IgG, IgM antibodies, and positive PCR to CMV infection, respectively. A systematic review was done by Mhandire and colleagues in 2019 to investigate the epidemiology of cytomegalovirus among pregnant women in Africa and retrieved 11 relevant original research papers. The prevalence of anti-CMV IgG and IgM antibodies ranged from 60 to 100% and 0-15.5%, respectively. While the prevalence of CMV DNA ranged from 0 to 29% [16]. Our results agreed also with the results of the study done by Zaki et al., 2016, in Mansura governorate in Egypt and revealed that 62.5%, 15%, and 8.3% of the pregnant women had positive IgG, IgM antibodies to CMV and positive PCR results [17]. On the other hand, Kamel et al., 2014 reported a higher prevalence of 100% and 7.3% for IgG and IgM, respectively [18]. Also, Porobic-Jahic et al., 2019, reported a high percentage of positive IgG antibodies to CMV in pregnant women, as much as 90.3%, while only 3% had positive IgM [19]. This difference in results may refer to the used serological method for screening and the different geographical locations.

In the current study, among 24 pregnant women who had positive IgM antibodies, only 15 had evidence of viral replication by positive CMV PCR (62.5%). This result closely agreed with Mohammad and Almosawi, 2014, who concluded that serological tests had a low diagnostic performance in identifying CMV infection in pregnant women. So, there are limitations to their interpretation that should be kept in mind [20].

In the present series, active CMV infection (evidenced by positive PCR and IgM) was more prominent in the group of pregnant females with chronic liver diseases not related to pregnancy than the other two groups. This may be related to the decreased immunity and subsequent susceptibility to CMV infection in these patients due to the presence of chronic liver disease. Primary CMV infection occurs early in life and is followed by a life-long persistence of the virus in a latent state, and reactivation may occur later in life, usually during periods of down-regulation of the immune system [21, 22]. Pregnancy with its downregulation of the immune system in addition to the chronic illness burden may explain the increased rate of current infection in this group of patients in our study.

Baseline and post-delivery mean values of LSM by ARFI in the current study were significantly higher among pregnant ladies with chronic liver diseases not related to pregnancy compared to the other two groups. Among this group with chronic liver disease, no significant difference regarding baseline and post-delivery mean ARFI was noted, this is due to the existence of liver injury by the presence of chronic liver disease in this group. Mueller and Sandrin, 2010, reported that all chronic liver diseases whether of toxic, genetic, autoimmune, or infectious origin undergo typical histological changes that ultimately lead to fibrosis/cirrhosis and excess deposition of the matrix that leads to increasing LS [23]. In either the normal pregnancy group or the group with liver disease associated with pregnancy in our study, mean ARFI results were significantly higher during pregnancy compared to the results after delivery, Ribeiro and colleagues 2019 found that liver stiffness and controlled attenuation parameters (CAP) values increase reversibly during normal pregnancies [10]. Ammon et al., 2018, reported that LS significantly increases in the third trimester despite normal pregnancy and rapidly normalizes after delivery. The rapid normalization after delivery is highly related to the release of mechanic pressure-related conditions of pregnancy and hemodynamic reasons. He also reported significantly higher LS in women with preeclampsia and ICP and considered it an independent predictor of complications with a rapid normalization after delivery [24].

In the current study, there was a negative significant correlation between ARFI results and CMV IgG and a positive significant correlation between ARFI results and CMV PCR within the group of ladies with normal pregnancy, which reflects the increase in liver stiffness with the increase in CMV viral load. Several studies had demonstrated an elevated LS in the absence of fibrosis [23, 25, 26] by conditions such as inflammation, cholestasis, congestion, elevated arterial pressure e.g. during exercise, rapid changes of the portal flow, food, and alcohol intake [27, 28].

In the current study, pregnancy outcomes significantly differed between pregnant with no CMV infection and those who had CMV infection. In pregnant with no CMV infection, abnormal pregnancy outcomes occurred in 9/95 (9.47%) pregnant women in the form of (four had preterm labor, three had a stillbirth, one delivered fetus with congenital anomalies, and one case of maternal mortality). In pregnant with CMV infection 20/106 (18.86%) pregnant women had an abnormal pregnancy outcome in the form of eleven preterm labor, four pregnant women delivered fetuses with congenital anomalies in form of congenital heart disease, one pregnant woman had an abortion, three pregnant women had a stillbirth, and one pregnant woman with maternal mortality). Fatima et al., 2017 showed that active CMV infection is one of the possible causes of bad obstetric outcomes [29].

Maternal mortality in the current study occurred only in pregnant females with chronic liver diseases not related to pregnancy (Two pregnant ladies (5.7%) died, one of them died due to severe hematemesis due to esophageal varices, while the second presented with acute HEV complicated by fulminant liver failure. Variceal bleeding most commonly occurs during the second and third trimesters when maternal blood volume is maximally expanded [30]. In a study done by Sowjanya and Sadha 2015, the incidence of hematemesis during pregnancy due to esophageal varices was 20 − 30% [31]. Acute variceal bleeding (AVB) has been also reported at the rate of 18–32% of pregnant women with cirrhosis by Britton 1982 [32]. Russell and Craigo 1998 reported much higher rates of variceal hemorrhage in women with known pre-existing varices, (up to 78%) with a quoted mortality rate of 18-50%, maybe due to a lack of effective screening programs for oesophageal varices at the time of their study [33].

Based on the results of the current study, we can conclude that CMV infection during pregnancy carries a significant risk for pregnant females with CLD. It should be kept in mind and screened in this cohort to avoid bad maternal and fetal outcomes.

## Data Availability

The data supporting the results are available from the corresponding author upon reasonable request.
